# Systematic analysis of lncRNA and microRNA dynamic features reveals diagnostic and prognostic biomarkers of myocardial infarction

**DOI:** 10.18632/aging.102667

**Published:** 2020-01-12

**Authors:** Hongbo Shi, Haoran Sun, Jiayao Li, Ziyi Bai, Jie Wu, Xiuhong Li, Yingli lv, Guangde Zhang

**Affiliations:** 1 College of Bioinformatics Science and Technology, Harbin Medical University, Harbin, Heilongjiang, China; 2 Laboratory of Medical Genetics, Harbin Medical University, Harbin, Heilongjiang, China; 3 Department of Cardiology, The Fourth Affiliated Hospital of Harbin Medical University, Harbin, Heilongjiang, China

**Keywords:** non-coding RNA, myocardial infarction, competing endogenous RNA, expression profile

## Abstract

Analyses of long non-coding RNAs (lncRNAs) and microRNAs (miRNAs) implicated in myocardial infarction (MI) have increased our understanding of gene regulatory mechanisms in MI. However, it is not known how their expression fluctuates over the different stages of MI progression. In this study, we used time-series gene expression data to examine global lncRNA and miRNA expression patterns during the acute phase of MI and at three different time points thereafter. We observed that the largest expression peak for mRNAs, lncRNAs, and miRNAs occurred during the acute phase of MI and involved mainly protein-coding, rather than non-coding RNAs. Functional analysis indicated that the lncRNAs and miRNAs most sensitive to MI and most unstable during MI progression were usually related to fewer biological functions. Additionally, we developed a novel computational method for identifying dysregulated competing endogenous lncRNA-miRNA-mRNA triplets (LmiRM-CTs) during MI onset and progression. As a result, a new panel of candidate diagnostic biomarkers defined by seven lncRNAs was suggested to have high classification performance for patients with or without MI, and a new panel of prognostic biomarkers defined by two lncRNAs evidenced high discriminatory capability for MI patients who developed heart failure from those who did not.

## INTRODUCTION

Myocardial infarction (MI) is one of the most severe coronary artery diseases, and a leading cause of morbidity and mortality in developed and developing countries [1]. Although currently available biomarkers such as cardiac troponins T and I and creatine kinase-MB are valuable aids in the diagnosis of MI, novel biomarkers may substantially increase early diagnosis accuracy to improve treatment strategies and patient outcomes. Additionally, since the 5-year mortality rate of the patients who developed heart failure (HF) after MI is as high as 50% [2], identifying early-stage prognostic biomarkers associated with post-MI HF is also very important. Although for some specific conditions the molecular mechanisms underlying MI have been defined, the dynamic modulation of gene expression, especially of non-coding RNAs (ncRNAs), during MI progression has not been fully investigated at a system level.

The proportion of the human genome encoding protein-coding genes is only ~2%, and estimations based on current sequencing methods suggest that most of the human transcriptome is composed of ncRNAs [3]. Long non-coding RNAs (lncRNAs) and microRNAs (miRNAs) are two important ncRNA classes. Many lncRNAs share miRNA binding sites with other coding and non-coding transcripts, and thus regulate the miRNA pool and influence posttranscriptional control by acting as competing endogenous RNAs (ceRNAs) [4, 5]. Moreover, mounting evidence indicates that lncRNAs and miRNAs can exert transcriptional and epigenetic regulation, and influence the course of various diseases including MI [6–9]. The potential of these ncRNAs as diagnostic and prognostic biomarkers in MI is suggested by studies demonstrating that dysregulated lncRNA and miRNA expression is closely associated with MI initiation and progression [10–12]. For example, inhibition of lncRNA-TUG1 was recently found to ameliorate myocardial injury and protect against acute MI by upregulation of miR-142-3p and subsequent suppression of HMGB1 and Rac1 expression [8].

Time-series gene expression data provide more valuable information than steady-state expression data for deciphering molecular mechanisms mediating biological processes and disease progression [13–17]. For example, dynamic gene regulatory networks for human myeloid differentiation were constructed using time-series RNA-seq and ATAC-seq data, from which a role for PU.1 and other key transcriptional regulators in maintaining and driving regulatory circuits specified during human myeloid differentiation was identified [18]. Whereas the dynamic characteristics of lncRNAs and circRNAs in cardiac differentiation have been recently explored [19] and our own work recently addressed the temporal changes in the expression, biological function, and regulatory interactions among miRNAs, TFs, and target genes during MI [20], the expression profiles of lncRNAs and miRNAs during MI progression remain unexplored.

To characterize dynamic patterns of lncRNA and miRNA expression and their potential functions in MI progression, we systematically analyzed lncRNA and miRNA expression profiles at four time points after MI. Additionally, we propose a novel algorithm for identifying dysregulated competing endogenous lncRNA-miRNA-mRNA triplets (LmiRM-CTs) during MI progression, with which we identified new panels of lncRNA biomarkers for MI diagnosis and prognosis.

## RESULTS

### Dynamic expression and functional characteristics of lncRNAs and miRNAs during MI progression

**Figure 1:**
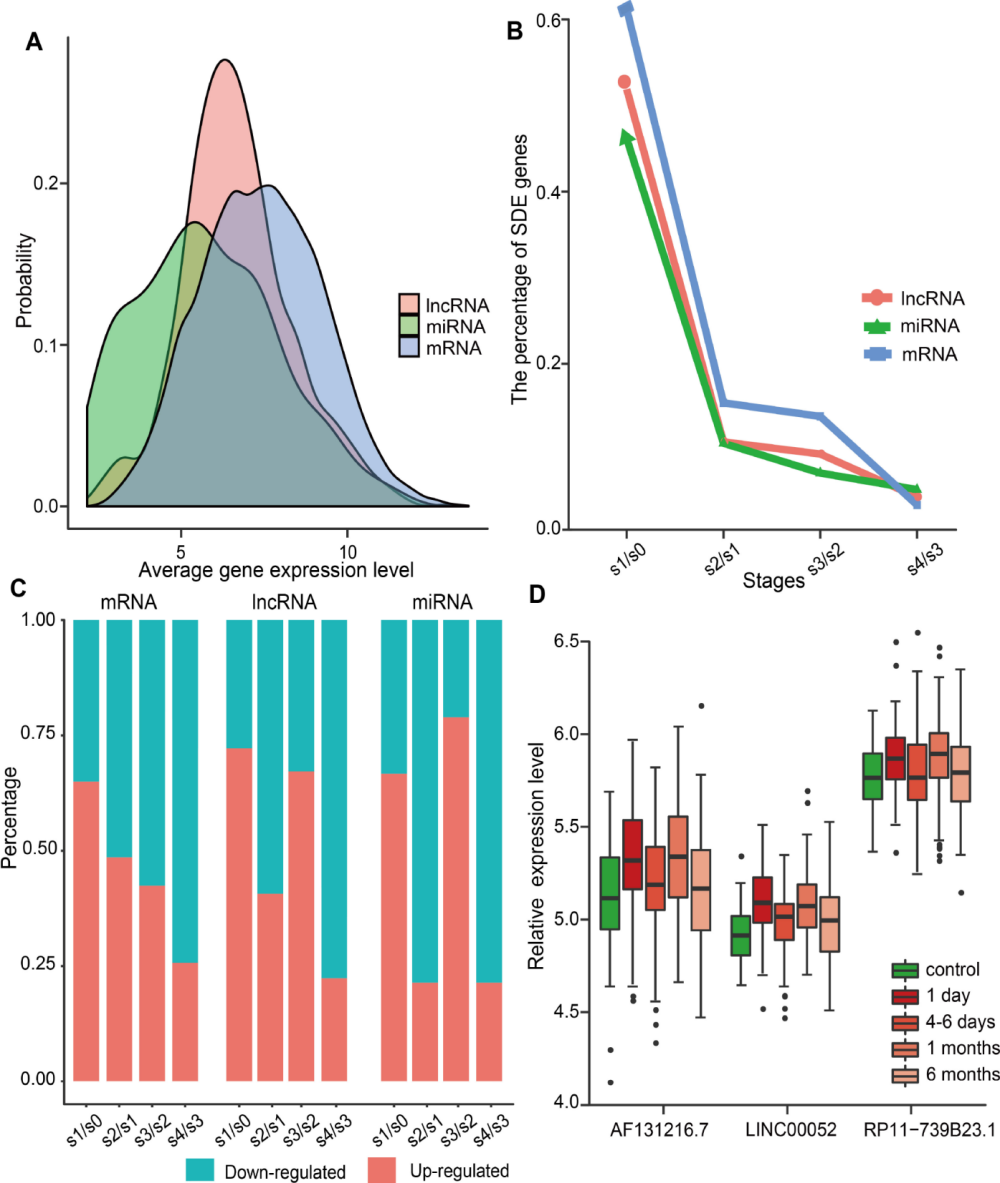
**Global gene expression distribution in MI and analysis of SDE genes during MI progression.** (**A**) Expression distribution of mRNAs, lncRNAs, and miRNAs in MI patients. (**B**) Percentage of SDE mRNAs, lncRNAs, and miRNAs estimated by comparing expression data between adjacent stages during MI progression. S0, control; S1, day 1 of MI; S2, 4-6 days after MI; S3, 1 month after MI; S4, 6 months after MI. (**C**) Percentage of upregulated and downregulated SDE genes during MI progression. (**D**) Relative expression levels of three lncRNAs that showed differential expression at each MI stage.

We investigated the dynamic expression of lncRNAs and miRNAs in MI patients at four time points: on admission (day 1 of MI), at discharge (4-6 days after MI), 1 month after MI, and 6 months after MI. To this end, we first analyzed the global expression distribution of mRNAs, lncRNAs, and miRNAs in MI patients. As shown in Figure 1A, the average expression level of miRNAs and lncRNAs was significantly lower than that of mRNAs (*p*<2.2e-16, Kolmogorov-Smirnov test), and miRNAs displayed the lowest expression levels. This was in accordance with current knowledge attesting lower expression of lncRNAs compared to protein-coding genes in different tissues [21]. Subsequently, we identified significantly differentially expressed (SDE) genes between adjacent MI stages, and calculated the percentage of SDE mRNAs, lncRNAs, and miRNAs relative to their total numbers in expression profiles (Figure 1B). Results showed that the largest percentage of SDE transcripts corresponded to the acute phase of MI (day 1), and among those, mRNAs were the most abundant. This indicated that the largest transcriptome change in MI occurs during the acute phase, and involves mainly differential expression of protein-coding, rather than ncRNA-coding, genes. In addition, we observed that most SDE transcripts (65.0% of mRNAs, 72.2% of lncRNAs, and 66.7% of miRNAs) were upregulated in the acute phase of MI (Figure 1C). Interestingly, while most SDE mRNAs were downregulated in the successive MI stages, the expression of SDE lncRNAs and miRNAs changed in parallel, as most of them were downregulated 4-6 days after MI, upregulated 1 month post-MI, and downregulated again 6 months after MI.

SDE lncRNAs and miRNAs were further explored to identify the most responsive ones at each stage. As a result, three lncRNAs (AF131216.7, LINC00052, and RP11-739B23.1), but no miRNAs, were retrieved. As shown in Figure 1D, the expression trend for these lncRNAs was consistent during MI progression. Whereas LINC00052 has been recently associated with certain cancers [7, 22, 23], no disease associations have been detected, to the best of our knowledge, for AF131216.7 and RP11-739B23.1.

**Figure 2:**
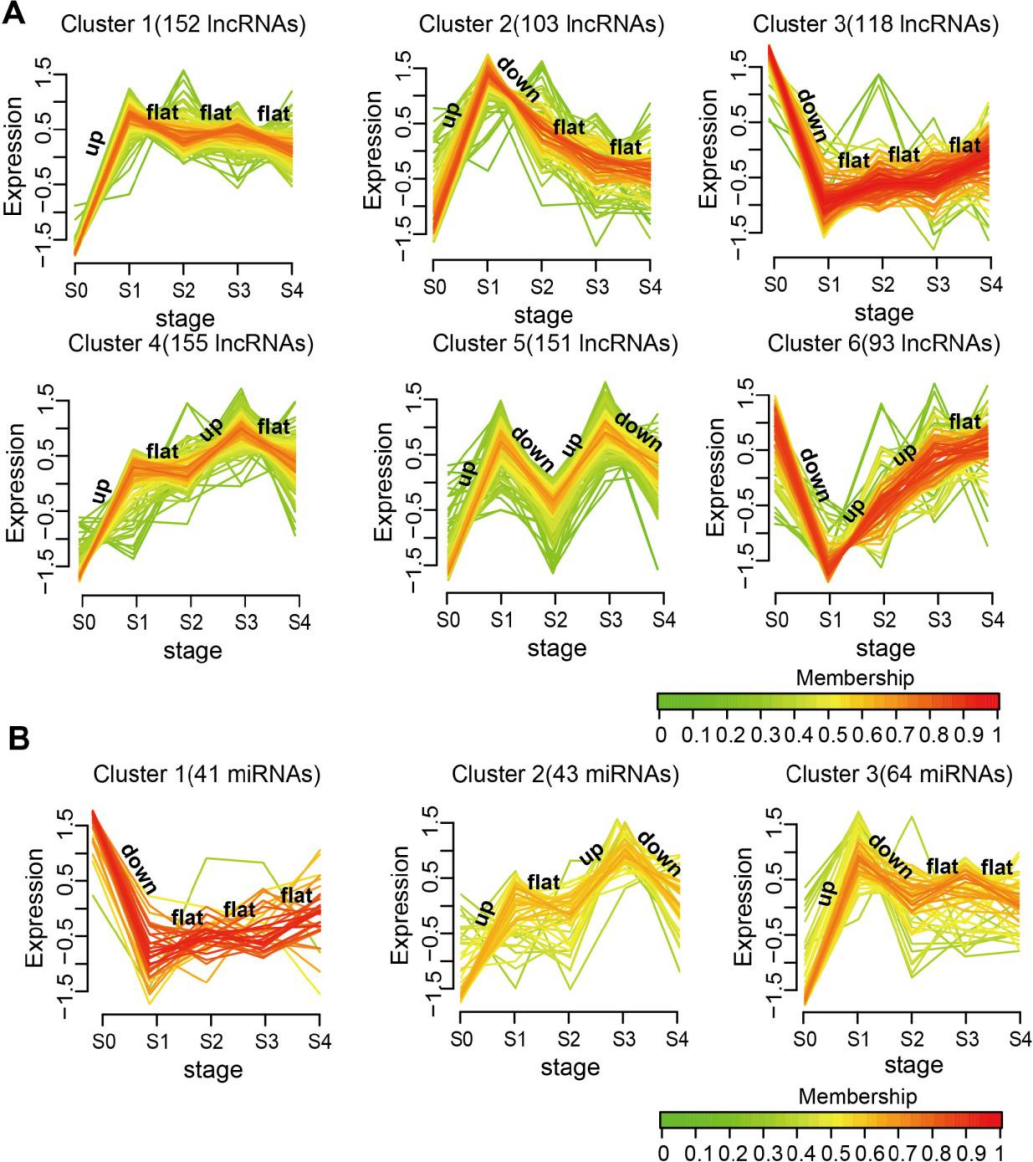
**Expression patterns of SDE lncRNAs and miRNAs during MI progression.** (**A**) SDE lncRNA clusters. (**B**) SDE miRNA clusters. The analysis was performed on R using the Mfuzz package.

To further study the dynamic changes in lncRNAs and miRNAs expression during MI progression, we classified stage-specific SDE lncRNAs and miRNAs into 6 and 3 clusters, respectively, using Mfuzz [24]. As shown in Figure 2A, lncRNAs in Clusters 1, 2,4, and 5 were all rapidly upregulated reaching a plateau on day 1 after MI, but they had different expression patterns in the following stages. Among all clusters, Cluster 5 had the most dramatic expression changes at each stage. Stage-specific variations were also noted for SDE miRNAs (Figure 2B). Cluster 1 was downregulated, while Clusters 2 and 3 were upregulated on day 1 after MI, and for these three clusters differing expression patterns ensued in subsequent stages. These results demonstrate that changes in the expression of lncRNAs and miRNAs during MI progression were not linear, but often evidenced drastic transitions at different time points.

**Figure 3:**
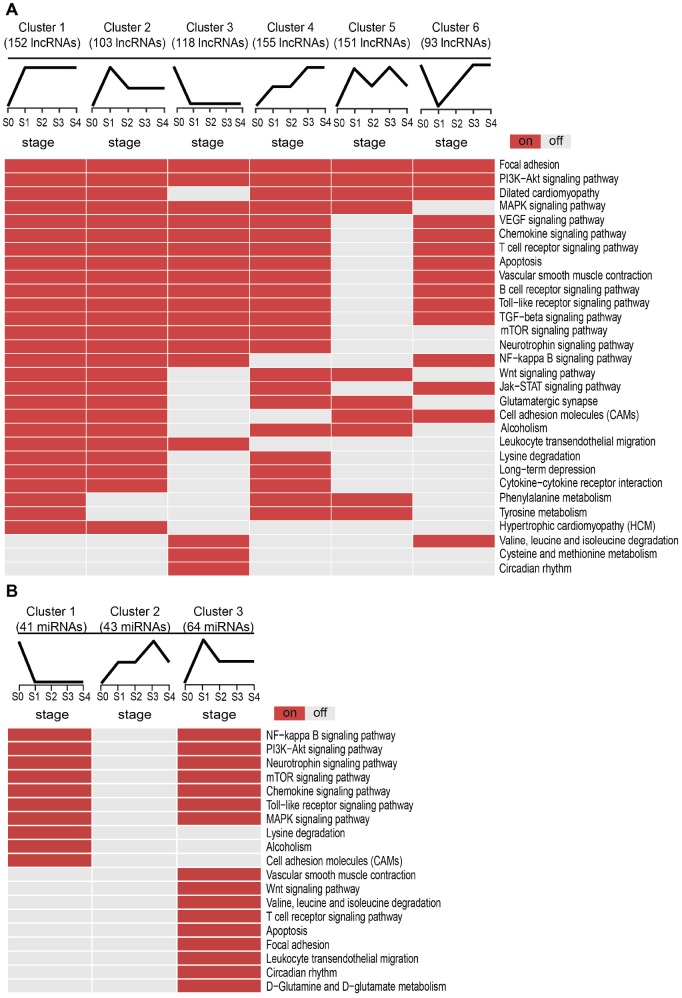
**Subpathway enrichment analysis of MI-related lncRNA/miRNA clusters.** (**A**) MI-related KEGG subpathways enriched for different lncRNA clusters. (**B**) MI-related KEGG subpathways enriched for different miRNA clusters.

Next, we examined the potential biological functions of the SDE lncRNAs and miRNAs included in the different clusters, and identified KEGG subpathways significantly enriched with these transcripts with basis on their co-expressed mRNAs (Supplementary Table 1). Subpathways associated with MI are shown in Figure 3. For lncRNAs (Figure 3A), Clusters 1 and 2 shared several common subpathways. Some of these, closely related to MI, such as PI3K-Akt signaling pathway, MAPK signaling pathway, Chemokine signaling pathway, T cell receptor signaling pathway, and apoptosis, were also shared with Clusters 3, 4, and 6. Simultaneously, we found that the lncRNAs and miRNAs with the sharpest expression changes during MI progression participated in fewer biological pathways. Thus, the lncRNAs in Cluster 5 enriched the fewest subpathways, while no subpathway was found to be significantly enriched with the miRNAs in Cluster 2 (Figure 3B). These results suggested that the lncRNAs and miRNAs most sensitive to environmental changes and thus unstable during MI progression might be of lesser biological relevance.

### Identification of dysregulated LmiRM-CTs and validation of their roles in MI

We developed a novel computational method for identifying dysregulated LmiRM-CTs in MI. This was done by integrating sample-matched expression profiles from 73 MI patients and 46 control samples in a Gene Expression Omnibus (GEO) dataset, and experimental verification of regulatory interactions among mRNAs, lncRNAs, and miRNAs (see Materials and Methods). As a result, 1,173 dysregulated LmiRM-CTs comprising 517 mRNAs, 49 lncRNAs, and 35 miRNAs were obtained (Supplementary Table 2). We validated the roles of these dysregulated LmiRM-CTs in MI from several perspectives and compared our method with the traditional one. The latter considered a LmiRM-CT as dysregulated when the mRNA, miRNA, and lncRNA in the LmiRM-CT satisfied the following criteria [25]: (1) they were all SDE in MI samples compared with controls; (2) the mRNA shared a significant number of miRNA binding sites with its paired lncRNA (hypergeometric test); (3) negative correlations existed within miRNA-mRNA and miRNA-lncRNA pairs, and the mRNA-lncRNA interaction was positively correlated in controls, but not in MI samples. Thus, the traditional method yielded 941 dysregulated LmiRM-CTs, which included 427 mRNAs, 46 lncRNAs, and 32 miRNAs (Supplementary Table 2), but did not provide scores for these LmiRM-CTs.

**Figure 4:**
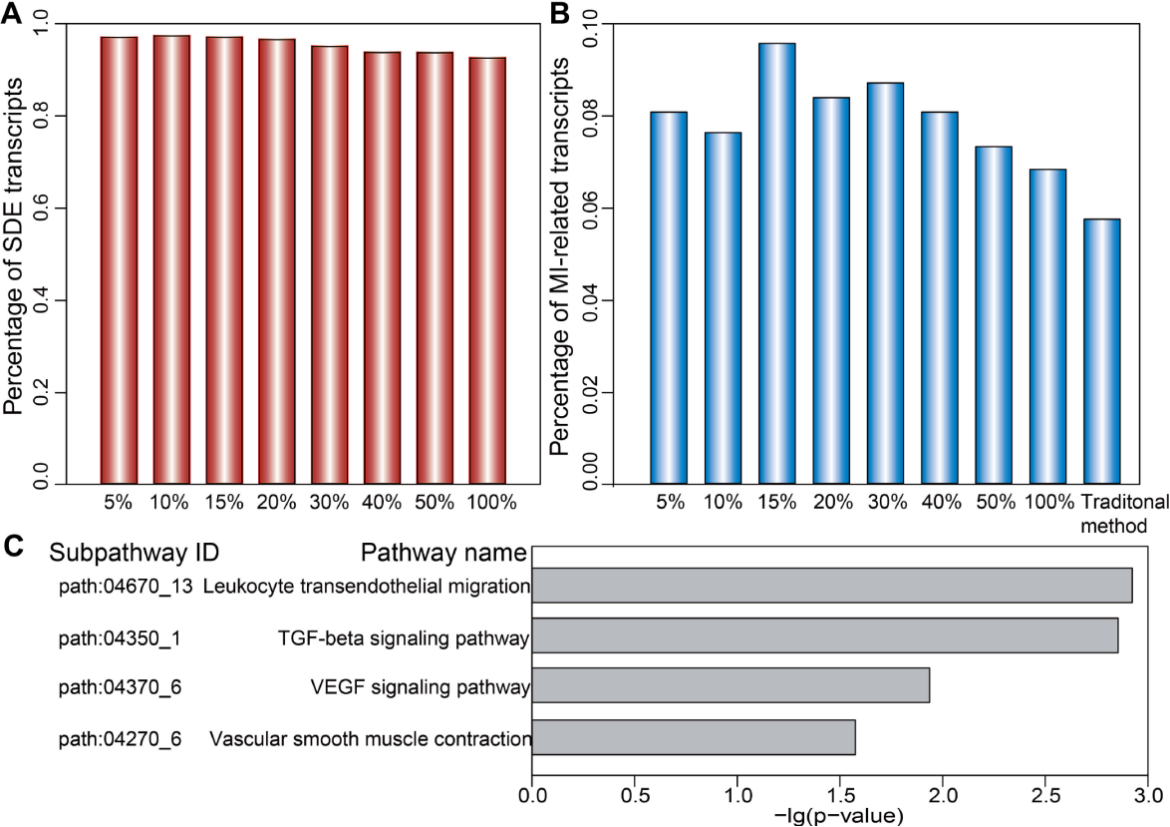
**Ranked distribution of SDE and MI-related transcripts.** Distribution of SDE transcripts (**A**) and MI-related transcripts (**B**) is shown for the top 5%, 10%, 15%, 20%, 30%, 40%, 50%, and 100% of dysregulated LmiRM-CTs. (**C**) Significantly enriched MI-related subpathways specifically detected by our method.

We first analyzed the distribution of SDE and MI-related mRNAs, miRNAs, and lncRNAs comprising dysregulated LmiRM-CTs. We found that the proportion of both SDE and MI-related transcripts in dysregulated LmiRM-CTs was significantly higher than in candidate LmiRM-CTs (hypergeometric test, *p*<0.001 and *p*<0.05, respectively). SDE and MI-related transcripts in the top 5%, 10%, 15%, 20%, 30%, 40%, and 50%, and in the full (100%) dysregulated LmiRM-CT set were also examined. As demonstrated in Figure 4A and 4B, and in Supplementary Table 3, the top-ranked dysregulated LmiRM-CTs had more SDE and MI-associated transcripts, while dysregulated LmiRM-CTs identified by the traditional method represented a lower rate of transcripts related to MI (5.74%) compared to our method (6.82%). Figure 4B shows that the top 15% dysregulated LmiRM-CTs exhibited the largest percentage of MI-related transcripts. Since ceRNAs analysis is still a developing field, newer studies on MI-associated ncRNAs aided by tools to increase transcript profiling accuracy might help unmask additional LmiRM-CTs of clinical relevance.

We next investigated the putative biological functions of the dysregulated LmiRM-CTs. Using SubpathwayMiner [26], 72 and 74 significant KEGG subpathways were revealed using our method and the traditional one, respectively (*p*<0.05, Supplementary Table 4). We found that several MI-related pathways, such as the signal transduction-related PI3K-Akt and MAPK pathway, the inflammation-related chemokine signaling pathway, and the immune-related B cell and T cell receptor signaling pathway were shared by both methods. However, some pathways with critical roles in MI initiation and progression, as those involving atherogenesis [27], inflammation-related leukocyte transendothelial migration, ventricular remodeling-related TGF-β signaling [28], blood pressure-related vascular smooth muscle contraction [29], and angiogenesis-related VEGF signaling, were significantly enriched on our method, but not on the traditional one (Figure 4C and Supplementary Table 4). These observations support the strength of our approach in identifying LmiRM-CTs dysregulated in MI.

### Progression-related dysregulated LmiRM-CTs analysis reveals diagnostic and prognostic biomarkers of MI

To identify potential diagnostic and prognostic biomarkers of MI, we applied our method to identify progression-related dysregulated LmiRM-CTs at the four indicated MI stages (Supplementary Table 5). Thus, 3,126 LmiRM-CTs (940 mRNAs, 59 miRNAs, and 77 lncRNAs) for day 1 of MI, 459 LmiRM-CTs (279 mRNAs, 36 miRNAs, and 55 lncRNAs) for days 4-6 after MI, 641 LmiRM-CTs (367 mRNAs, 45 miRNAs, and 58 lncRNAs) for month 1 after MI, and 749 LmiRM-CTs (427 mRNAs, 43 miRNAs, and 57 lncRNAs) for month 6 after MI were detected. The acute phase (day 1) of MI exhibited the largest number of dysregulated LmiRM-CTs, indicating that the most pronounced changes in gene expression and regulatory interactions occurred at this stage.

**Figure 5:**
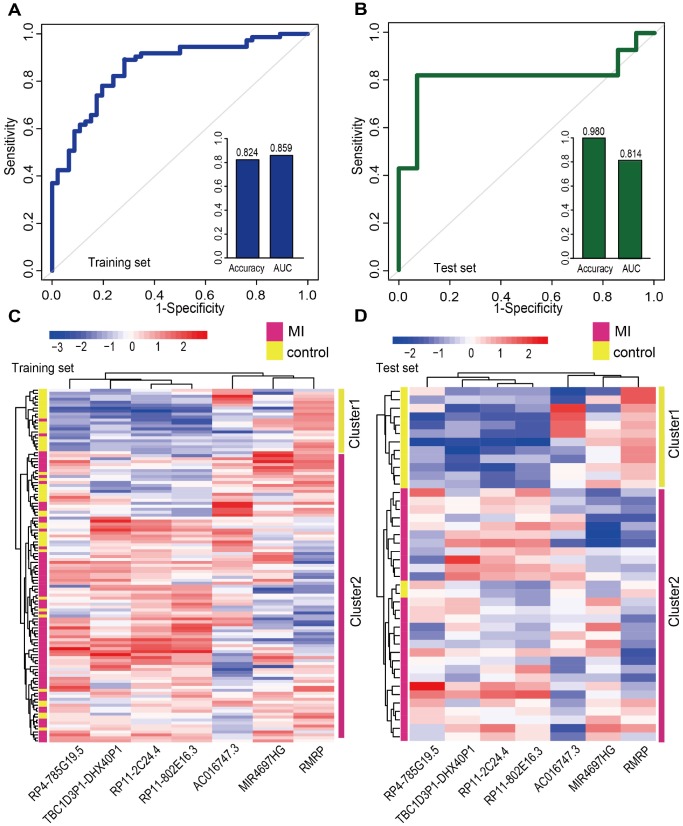
**Classification performance of diagnostic lncRNA biomarkers for MI.** Performance evaluation of the 7 diagnostic lncRNA biomarkers in the training (**A**) and test (**B**) sets using 5-fold cross-validation. Hierarchical clustering heat map of the expression profiles of 7 lncRNAs in (**C**) the training set (119 samples) and (**D**) the test set (42 samples).

We next focused on the 20 lncRNAs and 15 miRNAs specific for the MI acute phase (Supplementary Figure 1). A Random Forest supervised classification algorithm was applied and 10 lncRNAs and 7 miRNAs mostly related to MI occurrence were selected (see Materials and Methods). There were 2^10^-1=1023 and 2^7^-1=127 combinations of these lncRNAs and miRNAs, respectively. Classification accuracies for all the combinations were computed using the support vector machine (SVM) classification model, and the optimal biomarkers with the highest classification accuracy were identified. Consequently, two biomarker panels defined respectively by 7lncRNAs (AC016747.3, MIR4697HG, RMRP, RP11-2C24.4, RP11-802E16.3, RP4-785G19.5, and TBC1D3P1-DHX40P1) and 4 miRNAs (hsa-mir-144, hsa-mir-200b, hsa-mir-211, and hsa-mir-29a) were defined in the discovery cohort. In the training set, 5-fold cross-validation accuracies of 0.824 and 0.782 and AUC values of 0.859 and 0.823 were obtained, respectively, for the 7 lncRNAs and 4 miRNAs signatures (Figure 5A and Supplementary Figure 2A). Furthermore, we examined these signatures in an independent test set (GSE62646) which included 28 MI patients and 14 control samples. In this dataset, accuracies of 0.980 and 0.667 and AUC values of 0.814 and 0.700 were obtained for the 7 lncRNAs and 4 miRNAs signatures, respectively (Figure 5B and Supplementary Figure 2B). We next performed hierarchical clustering analysis using expression data of these two panel biomarkers and 2 major sample clusters were found (Figure 5C and 5D, and Supplementary Figure 2C and 2D). For the 7 lncRNAs, the rates of MI patients in the predicted MI group were 72.4% (71/98) and 93.3% (28/30) in the training and test sets, respectively, whereas the corresponding rates in the predicted control group were 90.5% (19/21) and 100% (12/12), respectively. The classification results of the 4 miRNAs were not better than those of the 7 lncRNAs (Supplementary Figure 2C and 2D).

**Figure 6:**
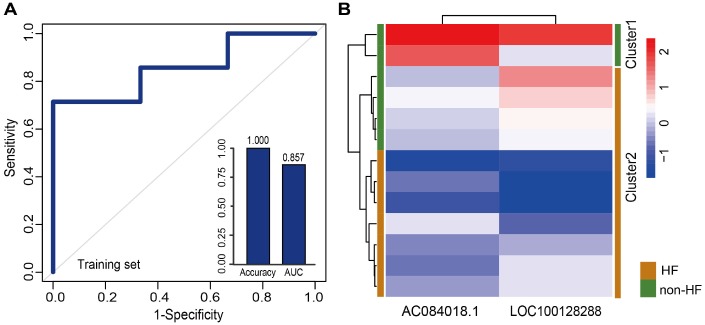
**Classification performance of prognostic lncRNA biomarkers for MI.** (**A**) Performance evaluation by 5-fold cross-validation of the 2 prognostic lncRNA biomarkers in the training set. (**B**) Hierarchical clustering heat map of 13 samples based on expression profiles of the 2 lncRNAs in the training set.

**Figure 7:**
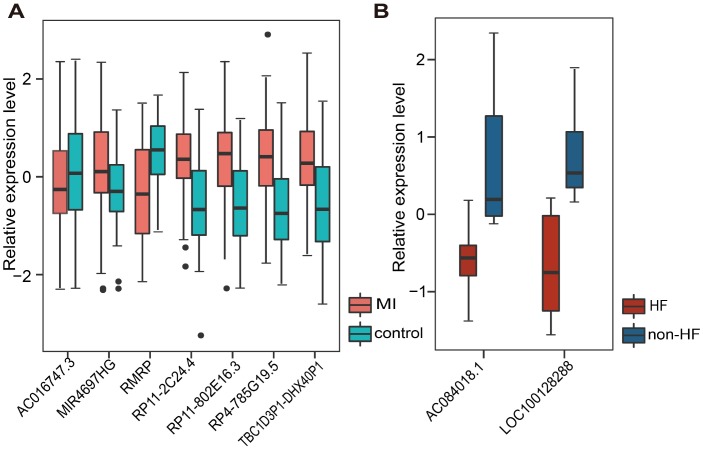
**Relative expression of lncRNA biomarkers for MI diagnosis and prognosis.** (**A**) Relative expression of 7 lncRNAs in MI and control samples. (**B**) Relative expression of 2 lncRNAs in HF and non-HF samples.

Additionally, we tested whether early changes in gene expression could predict disease prognosis and distinguish patients who developed HF after MI from those who did not. Therefore, 20 lncRNAs and 15 miRNAs that were specifically dysregulated in the acute phase of MI were examined. In the same way, 10 lncRNAs and 7 miRNAs mostly related to MI prognosis were selected. It is worth noting that they were different from those associated with MI diagnosis. Finally, two panel biomarkers defined by 2 lncRNAs (AC084018.1 and LOC100128288) and 2 miRNAs (hsa-mir-211 and hsa-mir-214) were defined, showing respectively accuracies of 1.000 and 0.385 and AUC values of 0.857 and 0.857 using 5-fold cross-validation (Figure 6A and Supplementary Figure 3A). Hierarchical clustering heat maps of the two biomarker panels are shown in Figure 6B and Supplementary Figure 3B. For the 2 lncRNAs, HF patient rate in the predicted HF group was 63.6% (7/11), whereas the corresponding rate in the predicted non-HF group was 100% (2/2). The classification results of the 2 miRNAs were no better than those of the 2 lncRNAs. The above results suggest that the lncRNA biomarkers we identified had higher classification efficiency than the miRNA biomarkers for both MI diagnosis and prognosis, and that the lncRNA signatures reliably distinguished MI patients from controls and MI patients who developed HF from those who did not. Additionally, we found that most MI diagnostic lncRNA and miRNA biomarkers were upregulated in MI patients, while all the MI prognostic lncRNA and miRNA biomarkers were downregulated in MI patients who developed HF (Figure 7 and Supplementary Figure 4).

## DISCUSSION

The current study explored the global dynamic expression and tentative functions of lncRNAs and miRNAs during MI progression by systematically analyzing MI-related time-series gene expression data. Using a novel computational method integrating sample-matched mRNA, lncRNA, and miRNA expression profiles, significantly dysregulated ceRNA triplets were identified at different stages of MI progression.

We obtained lncRNA and miRNA expression profile data for dynamic expression analysis through microarray re-annotation. Evidence shows that about 10% to 30% of probes in microarrays designed for protein-coding genes actually map to ncRNAs [30], thus acquisition of lncRNA expression information through re-annotation is a widely used approach in transcriptomics studies [31, 32]. According to a previously described pipeline [33], we extracted expression data of lncRNAs and miRNAs directly from existing expression profiles, and thus reduced the error. As a result, 1,282 lncRNAs and 260 miRNAs were acquired. Currently, there are 1,913 miRNAs in the miRBase (release 22) database and 15,779 lncRNAs in GENCODE (release 28). The ratio of miRNAs (13.6%) we obtained from re-annotation was larger than that of lncRNAs (8.12%).

We first retrieved candidate LmiRM-CTs from control samples (patients with stable coronary artery disease and without a history of myocardial infarction) and assumed that their dysfunction was associated with the occurrence and development of MI. The extent of dysfunction of a LmiRM-CT was assessed by integrating differential expression of the corresponding transcripts and differential linked co-expression between two transcripts in two biological states (consecutive MI stages). Therefore, the method not only reflects expression changes of a single transcript, but also reveals concurrent changes between two transcripts. Compared with the traditional method, our method detected a larger number of dysregulated LmiRM-CTs, and expanded the number of MI-related biological pathways enriched by these ceRNA triplets. Because our method was based on sample-matched mRNA, miRNA, and lncRNA expression profiles with experimentally verified interactions within individual miRNA-mRNA and miRNA-lncRNA duplexes, superior analysis stringency was achieved. Interestingly, SVM classification analysis suggested that two separate lncRNA signatures reliably distinguished MI patients from controls and MI patients who developed HF from those who did not. Information for these lncRNAs is scarce or absent, especially on relation to MI. Among the dysregulated lncRNAs, RMRP was recently found to be upregulated by hypoxia in cardiomyocytes, and to aggravate myocardial ischemia-reperfusion injury by sponging miR-206 to target ATG3 expression [34]. In addition, upregulation of RMRP was also reported to promote the activation of cardiac fibroblasts by regulating miR-613 [7].

In conclusion, the present study provided a system level exploration of the landscape of dysregulated lncRNAs and miRNAs, and their interactions with differentially expressed mRNAs, at different time points over MI progression. Using a novel computational approach, we present two new lncRNA biomarker panels to aid MI diagnosis and predict HF in MI patients.

Worth noting, several limitations stand out in the present work and merit further experimental validation. These include underrepresentation of the actual number of miRNAs and lncRNAs extractable from expression profiles, incomplete or biased characterization of cell- tissue- or disease-specific regulatory interactions between miRNA-mRNA and miRNA-lncRNA related to MI, and the relatively small sampling size of the GEO dataset examined in this study.

## MATERIALS AND METHODS

### RNA expression profiling

The mRNA expression profile data of GSE59867 (based on Affymetrix Human Gene 1.0 ST Array) was downloaded from the GEO database (https://www.ncbi.nlm.nih.gov/geo/query/acc.cgi?acc=gse59867) [35]. The dataset included 111 patients with ST-segment elevation MI and a control group comprised of 46 patients with stable coronary artery disease and without a history of MI. The corresponding mRNA expression profiles were obtained from peripheral blood mononuclear cells at four time points: on admission (MI day 1), at discharge (4-6 days after MI), 1 month after MI, and 6 months after MI. We retained samples that included expression profiles from all four time points, and 73 case samples and 46 controls were obtained. Additionally, among the 73 patients 13 had follow-up data, including 7 HF and 6 non-HF cases.

LncRNA expression profiles were obtained by applying a lncRNA classification pipeline based on transcript clusters in the Affymetrix 1.0 ST Arrays [33]. First, we downloaded the Annotation file (HuGene-1_0-st-v1 Transcript Cluster Annotations, CSV, Release 36) and then mapped gene names to associated Affymetrix 1.0 ST transcript cluster IDs. Each transcript cluster was assigned to an mRNA transcript associated with an Ensembl gene ID and/or RefSeq transcript ID. Second, for transcript clusters with Ensembl gene IDs, we retained those annotated as “lincRNA”, “antisense_RNA”, “processed_transcript”, “sense_intronic”, “TEC”, “3prime_overlapping_ncRNA”, “bidirectional_promoter_lncRNA”, “sense_overlapping” and “non_coding” in the GENCODE project (https://www.gencodegenes.org/human/release_28.html) [3]. For transcript clusters with RefSeq transcript IDs, those labeled as “NR_” (non-coding RNA) were retained. Finally, duplicated transcripts were removed and lncRNA expression profiles were determined from 1,655 transcript clusters and 1,282 lncRNAs. MiRNA expression profiles were obtained using the same pipeline as for lncRNAs. Transcript clusters with an mRNA-assignment that included an miRNA and/or Refseq transcript ID were retained. We unified miRNA names using miRBase (release 22) database, and miRNA expression profiles comprising 252 transcript clusters and 260 miRNAs were acquired.

### Expression profile analyses

For multiple probes corresponding to the same gene within the mRNA, lncRNA, and miRNA expression profiles, their median value was taken as expression value for such gene. We then retained protein-coding genes in mRNA expression profiles. Finally, 18,451 mRNAs, 1,282 lncRNAs, and 260 miRNAs were retained for further analysis. SDE genes were screened by comparing expression data between two adjacent stages, and differential analysis was performed using an empirical Bayesian method implemented in R “limma” package [36]. Genes with *p*<0.05 were selected as SDE genes.

### Expression and functional analysis of lncRNAs and miRNAs

LncRNAs and miRNAs were grouped according to their dynamic expression patterns. The R package “Mfuzz” [24] was used to detect lncRNA/miRNA clusters with consistent expression trends during MI progression. To investigate their biological functions, co-expressed mRNAs were extracted based on a Pearson correlation coefficient (PCC)>0.7 or <-0.7 and *p*<0.01. KEGG sub-pathway enrichment analysis for co-expressed mRNAs was implemented using the R “SubpathwayMiner” package [26]. Significantly enriched sub-pathways were identified based on *p*<0.05. If multiple significantly enriched sub-pathways corresponded to an entire pathway, the sub-pathway with the lowest *p* value was retained.

### Regulatory interactions between miRNA-mRNA and miRNA-lncRNA duplexes

Experimentally verified miRNA-mRNA regulatory relationships were collected from TarBase (version 6.0) [37], miRTarBase (version 7.0) [38], and miRecords (version 4) [39] databases, and 391,694 non-redundant miRNA-mRNA interactions were obtained. Experimentally confirmed miRNA-lncRNA interactions were retrieved from starBase v2.0 [40] and DIANA-LncBase v2.0 [41] databases, and 64,716 non-redundant miRNA-lncRNA relationships were retained.

### Candidate LmiRM-CTs

We assumed that LmiRM-CTs existed in control samples, and their dysfunction would lead to initiation and progression of cardiac diseases. Based on the ceRNA hypothesis [4, 5], a candidate LmiRM-CT in control samples was identified if it met all of the following criteria: (1) the mRNA and the lncRNA shared a significant number of miRNAs as determined by a hypergeometric test (*p*<0.05); (2) the PCC of the mRNA (lncRNA) and the miRNA was negatively correlated (*p*<0.05), and the PCC of the lncRNA and the mRNA was positively correlated (*p*<0.05). To increase the reliability of the results, we retained the top correlated interaction pairs for further analysis [42, 43]. Interaction pairs with PCCs above the threshold of the 90^th^ percentile of the corresponding overall correlation distribution were retained. By integrating expression profile data in the control group and confirmed miRNA-mRNA and miRNA-lncRNA interactions, 7,468 candidate LmiRM-CTs comprising 120 lncRNAs, 97 miRNAs, and 1,718 mRNAs were retained.

### Identification of dysregulated LmiRM-CTs in MI

Dysregulated LmiRM-CTs in MI were identified by considering the dysregulation extent of all genes (nodes) and their regulatory/competing relationships (edges) in two different biological states (i.e. case and control samples) using-sample matched expression profiles. First, each node score was calculated through the following formulas according to the extent of differential expression [44, 45]:

$$Score_{node}=\varphi^{-1}(1-2\times (1-\varphi (D_{node})))$$ (1)

$$D_{node}=(-\log_{10}p)\;\cdot |\log_{2}FC|$$ (2)

where *φ*^−1^ is the inverse normal cumulative distribution function, *p* is the *p*-value reflecting the significance of differential expression determined by the R ‘limma’ package, and *FC* is the corresponding fold expression change. Second, each edge score was computed according to the change ingene co-expression between two different biological states [45–47] using equations (3) and (4):

$$Score_{edge}=\varphi^{-1}(1-2\times (1-\varphi (|\xi |)))$$ (3)

$$\xi =\frac{F(r_{state2})\cdot (-\log_{10}p_{state2})-F(r_{state1})\cdot (-\log_{10}p_{state1})}{\sqrt{\frac{1}{n_{state2}-3}+\frac{1}{n_{state1}-3}}}$$ (4)

$$F(r)=\frac{1}{2}(\ln\frac{1+r}{1-r})$$ (5)

Here, *r_state_*_2_ and *r_state_*_1_ are the PCCs of gene expression in *state*_2_ and *state*_1_ samples (i.e. case and control samples), respectively, *p_state_*_2_ and *p_state_*_1_ are their respective *p*-values, and *n_state_*_2_ and *n_state_*_1_ are the corresponding number of samples. *F* is the Fisher transformation function, applied to improve the power of identifying differentially rewired genes [48]. Finally, the score of candidate LmiRM-CTs was computed by integrating the node and the edge scores:

$$\begin{array}{l} Score=\omega \frac{\sum_{\text{node}\in LmiRM-CT}Score_{node}}{\sqrt{n_{node}}}\\ \;\;\;\;\;\;\;\;\;+(1-\omega )\frac{\sum_{\text{edge}\in LmiRM-CT}Score_{\text{edge}}}{\sqrt{n_{edge}}} \end{array}$$ (6)

where *n_node_* and *n_edge_* are the number of nodes and edges in the LmiRM-CT. Here, it is considered that regulatory relationships exist for both miRNA-lncRNA and miRNA-mRNA duplexes, and competing relationships exist between lncRNA-mRNA pairs. Therefore, a value of 3 is assigned to both *n_node_* and *n_edge_*. The weight parameter ω (0≤ω≤1) is used to control the contribution of the node and edge scores. Here, both scores were considered equally weighted, and *ω* was defined as 0.5.

We performed permutation analysis to evaluate the significance of a given LmiRM-CT. An arbitrary LmiRM-CT was generated by randomly selecting a lncRNA, an miRNA, and an mRNA, and its score calculated through the above equations. This process was repeated 10,000 times, and the empirical *p*-value was defined as the proportion of randomly obtained scores larger than the observed score:

$$p-value=(\text{Number}\;\text{of}\;Score_{random}>Score)\text{/}10000$$ (7)

LmiRM-CTs with *p*<0.05 were selected as dysregulated LmiRM-CTs.

### Collection of mRNAs, lncRNAs, and miRNAs related to MI

MI-related mRNAs were collected from DisGeNET (V5.0) [49], a comprehensive human gene-disease association database that integrates many current, widely used gene-disease databases such as OMIM [50], the Genetic Association Database (GAD) [51], the Comparative Toxico genomics Database (CTD) [52], the Mouse Genome Database (MGD) [53], PubMed, and Uniprot [54]. We removed repeated gene-disease entries, and 990 non-redundant MI-related mRNAs were acquired.

MI-related lncRNAs were collected by performing a comprehensive literature review. Relevant articles were compiled from a experimentally confirmed human lncRNA-disease association database, LncRNADisease (version 2.0) [6] using the search phase “myocardial infarction”, and from PubMed using the search phrase “myocardial infarction AND (lncRNA OR long non-coding RNA)”. Each article was manually searched for lncRNAs with aberrant expression in MI. Finally, 37 unique lncRNAs were obtained.

MI-related miRNAs were collected from a manually curated and experimentally confirmed human miRNA-disease association database, HMDD (version 3.1) [55]. After removing redundant miRNA-disease relations and unifying miRNA names according to the miRBase database (release 22) [56], 101 miRNAs were selected.

### Identification of candidate diagnostic and prognostic biomarkers for MI

Candidate diagnostic and prognostic biomarkers for MI were identified by applying a classification model based on SVM. This process was performed using the R ‘e1071’ package, and the performance was estimated by classification accuracy and the area under the receiver operating characteristic curve (AUC) based on 5-fold cross-validation. AUC values range from 0 to 1, with 0.5 indicating random performance and 1.0 implying perfect predictive performance.

LncRNAs and miRNAs highly related to MI diagnosis and prognosis were selected using a Random Forest supervised classification algorithm [57]. At each step, an importance score was computed for each lncRNA/miRNA using the out-of-bag samples via permutation test, and the lowest scoring thirds of the lncRNAs/miRNAs were removed. We then reserved certain lncRNAs/miRNAs considering a balance between classification accuracy and the number of lncRNAs/miRNAs. Finally, classification accuracy for all combinations of the remaining lncRNAs/miRNAs was assessed using SVM, and the optimal lncRNA/miRNA biomarkers were obtained.

## Supplementary Material

Supplementary Figures

Supplementary Table 1

Supplementary Table 2

Supplementary Table 3

Supplementary Table 4

Supplementary Table 5

## References

[1] Mozaffarian D, Benjamin EJ, Go AS, Arnett DK, Blaha MJ, Cushman M, de Ferranti S, Després JP, Fullerton HJ, Howard VJ, Huffman MD, Judd SE, Kissela BM, et al, and American Heart Association Statistics Committee and Stroke Statistics Subcommittee. Heart disease and stroke statistics—2015 update: a report from the American Heart Association. Circulation. 2015; 131:e29–322. 10.1161/CIR.0000000000000152 25520374

[2] Roger VL, Weston SA, Redfield MM, Hellermann-Homan JP, Killian J, Yawn BP, Jacobsen SJ. Trends in heart failure incidence and survival in a community-based population. JAMA. 2004; 292:344–50. 10.1001/jama.292.3.344 15265849

[3] Birney E, Stamatoyannopoulos JA, Dutta A, Guigó R, Gingeras TR, Margulies EH, Weng Z, Snyder M, Dermitzakis ET, Thurman RE, Kuehn MS, Taylor CM, Neph S, et al, and Children’s Hospital Oakland Research Institute. Identification and analysis of functional elements in 1% of the human genome by the ENCODE pilot project. Nature. 2007; 447:799–816. 10.1038/nature05874 17571346 PMC2212820

[4] Salmena L, Poliseno L, Tay Y, Kats L, Pandolfi PP. A ceRNA hypothesis: the Rosetta Stone of a hidden RNA language? Cell. 2011; 146:353–58. 10.1016/j.cell.2011.07.014 21802130 PMC3235919

[5] Tay Y, Rinn J, Pandolfi PP. The multilayered complexity of ceRNA crosstalk and competition. Nature. 2014; 505:344–52. 10.1038/nature12986 24429633 PMC4113481

[6] Bao Z, Yang Z, Huang Z, Zhou Y, Cui Q, Dong D. LncRNADisease 2.0: an updated database of long non-coding RNA-associated diseases. Nucleic Acids Res. 2019; 47:D1034–37. 10.1093/nar/gky905 30285109 PMC6324086

[7] Zhang SY, Huang SH, Gao SX, Wang YB, Jin P, Lu FJ. Upregulation of lncRNA RMRP promotes the activation of cardiac fibroblasts by regulating miR-613. Mol Med Rep. 2019; 20:3849–57. 10.3892/mmr.2019.10634 31485650 PMC6755198

[8] Zhou M, Zhao H, Wang X, Sun J, Su J. Analysis of long noncoding RNAs highlights region-specific altered expression patterns and diagnostic roles in Alzheimer’s disease. Brief Bioinform. 2019; 20:598–608. 10.1093/bib/bby021 29672663

[9] Zhou M, Hu L, Zhang Z, Wu N, Sun J, Su J. Recurrence-Associated Long Non-coding RNA Signature for Determining the Risk of Recurrence in Patients with Colon Cancer. Mol Ther Nucleic Acids. 2018; 12:518–29. 10.1016/j.omtn.2018.06.007 30195788 PMC6076224

[10] Vausort M, Wagner DR, Devaux Y. Long noncoding RNAs in patients with acute myocardial infarction. Circ Res. 2014; 115:668–77. 10.1161/CIRCRESAHA.115.303836 25035150

[11] Li L, Wang L, Li H, Han X, Chen S, Yang B, Hu Z, Zhu H, Cai C, Chen J, Li X, Huang J, Gu D. Characterization of LncRNA expression profile and identification of novel LncRNA biomarkers to diagnose coronary artery disease. Atherosclerosis. 2018; 275:359–67. 10.1016/j.atherosclerosis.2018.06.866 30015300

[12] Li H, Chen C, Fan J, Yin Z, Ni L, Cianflone K, Wang Y, Wang DW. Identification of cardiac long non-coding RNA profile in human dilated cardiomyopathy. Cardiovasc Res. 2018; 114:747–58. 10.1093/cvr/cvy012 29365080

[13] Bar-Joseph Z, Gitter A, Simon I. Studying and modelling dynamic biological processes using time-series gene expression data. Nat Rev Genet. 2012; 13:552–64. 10.1038/nrg3244 22805708

[14] Turner M. Is transcription the dominant force during dynamic changes in gene expression? Adv Exp Med Biol. 2011; 780:1–13. 10.1007/978-1-4419-5632-3_1 21842360

[15] Port JD, Walker LA, Polk J, Nunley K, Buttrick PM, Sucharov CC. Temporal expression of miRNAs and mRNAs in a mouse model of myocardial infarction. Physiol Genomics. 2011; 43:1087–95. 10.1152/physiolgenomics.00074.2011 21771878 PMC3217325

[16] Reinsbach S, Nazarov PV, Philippidou D, Schmitt M, Wienecke-Baldacchino A, Muller A, Vallar L, Behrmann I, Kreis S. Dynamic regulation of microRNA expression following interferon-γ-induced gene transcription. RNA Biol. 2012; 9:978–89. 10.4161/rna.20494 22767256

[17] Nazarov PV, Reinsbach SE, Muller A, Nicot N, Philippidou D, Vallar L, Kreis S. Interplay of microRNAs, transcription factors and target genes: linking dynamic expression changes to function. Nucleic Acids Res. 2013; 41:2817–31. 10.1093/nar/gks1471 23335783 PMC3597666

[18] Ramirez RN, El-Ali NC, Mager MA, Wyman D, Conesa A, Mortazavi A. Dynamic Gene Regulatory Networks of Human Myeloid Differentiation. Cell Syst. 2017; 4:416–29.e3. 10.1016/j.cels.2017.03.005 28365152 PMC5490374

[19] Li Y, Zhang J, Huo C, Ding N, Li J, Xiao J, Lin X, Cai B, Zhang Y, Xu J. Dynamic Organization of lncRNA and Circular RNA Regulators Collectively Controlled Cardiac Differentiation in Humans. EBioMedicine. 2017; 24:137–46. 10.1016/j.ebiom.2017.09.015 29037607 PMC5652025

[20] Shi H, Zhang G, Wang J, Wang Z, Liu X, Cheng L, Li W. Studying Dynamic Features in Myocardial Infarction Progression by Integrating miRNA-Transcription Factor Co-Regulatory Networks and Time-Series RNA Expression Data from Peripheral Blood Mononuclear Cells. PLoS One. 2016; 11:e0158638. 10.1371/journal.pone.0158638 27367417 PMC4930172

[21] Jiang C, Li Y, Zhao Z, Lu J, Chen H, Ding N, Wang G, Xu J, Li X. Identifying and functionally characterizing tissue-specific and ubiquitously expressed human lncRNAs. Oncotarget. 2016; 7:7120–33. 10.18632/oncotarget.6859 26760768 PMC4872773

[22] Yu G, Xiong D, Liu Z, Li Y, Chen K, Tang H. Long noncoding RNA LINC00052 inhibits colorectal cancer metastasis by sponging microRNA-574-5p to modulate CALCOCO1 expression. J Cell Biochem. 2019; 120:17258–72. 10.1002/jcb.28988 31104316

[23] Xiong X, Shi Q, Yang X, Wang W, Tao J. LINC00052 functions as a tumor suppressor through negatively modulating miR-330-3p in pancreatic cancer. J Cell Physiol. 2019; 234:15619–26. 10.1002/jcp.28209 30712321

[24] Kumar L, E Futschik M. Mfuzz: a software package for soft clustering of microarray data. Bioinformation. 2007; 2:5–7. 10.6026/97320630002005 18084642 PMC2139991

[25] Liang H, Yu T, Han Y, Jiang H, Wang C, You T, Zhao X, Shan H, Yang R, Yang L, Shan H, Gu Y. LncRNA PTAR promotes EMT and invasion-metastasis in serous ovarian cancer by competitively binding miR-101-3p to regulate ZEB1 expression. Mol Cancer. 2018; 17:119. 10.1186/s12943-018-0870-5 30098599 PMC6087007

[26] Li C, Li X, Miao Y, Wang Q, Jiang W, Xu C, Li J, Han J, Zhang F, Gong B, Xu L. SubpathwayMiner: a software package for flexible identification of pathways. Nucleic Acids Res. 2009; 37:e131. 10.1093/nar/gkp667 19706733 PMC2770656

[27] Bhui R, Hayenga HN. An agent-based model of leukocyte transendothelial migration during atherogenesis. PLOS Comput Biol. 2017; 13:e1005523. 10.1371/journal.pcbi.1005523 28542193 PMC5444619

[28] Ellmers LJ, Scott NJ, Medicherla S, Pilbrow AP, Bridgman PG, Yandle TG, Richards AM, Protter AA, Cameron VA. Transforming growth factor-beta blockade down-regulates the renin-angiotensin system and modifies cardiac remodeling after myocardial infarction. Endocrinology. 2008; 149:5828–34. 10.1210/en.2008-0165 18653707

[29] Touyz RM, Alves-Lopes R, Rios FJ, Camargo LL, Anagnostopoulou A, Arner A, Montezano AC. Vascular smooth muscle contraction in hypertension. Cardiovasc Res. 2018; 114:529–39. 10.1093/cvr/cvy023 29394331 PMC5852517

[30] Risueño A, Fontanillo C, Dinger ME, De Las Rivas J. GATExplorer: genomic and transcriptomic explorer; mapping expression probes to gene loci, transcripts, exons and ncRNAs. BMC Bioinformatics. 2010; 11:221. 10.1186/1471-2105-11-221 20429936 PMC2875241

[31] Du Z, Fei T, Verhaak RG, Su Z, Zhang Y, Brown M, Chen Y, Liu XS. Integrative genomic analyses reveal clinically relevant long noncoding RNAs in human cancer. Nat Struct Mol Biol. 2013; 20:908–13. 10.1038/nsmb.2591 23728290 PMC3702647

[32] Zhou M, Zhao H, Wang Z, Cheng L, Yang L, Shi H, Yang H, Sun J. Identification and validation of potential prognostic lncRNA biomarkers for predicting survival in patients with multiple myeloma. J Exp Clin Cancer Res. 2015; 34:102. 10.1186/s13046-015-0219-5 26362431 PMC4567800

[33] Zhang X, Sun S, Pu JK, Tsang AC, Lee D, Man VO, Lui WM, Wong ST, Leung GK. Long non-coding RNA expression profiles predict clinical phenotypes in glioma. Neurobiol Dis. 2012; 48:1–8. 10.1016/j.nbd.2012.06.004 22709987

[34] Kong F, Jin J, Lv X, Han Y, Liang X, Gao Y, Duan X. Long noncoding RNA RMRP upregulation aggravates myocardial ischemia-reperfusion injury by sponging miR-206 to target ATG3 expression. Biomed Pharmacother. 2019; 109:716–25. 10.1016/j.biopha.2018.10.079 30551524

[35] Maciejak A, Kiliszek M, Michalak M, Tulacz D, Opolski G, Matlak K, Dobrzycki S, Segiet A, Gora M, Burzynska B. Gene expression profiling reveals potential prognostic biomarkers associated with the progression of heart failure. Genome Med. 2015; 7:26. 10.1186/s13073-015-0149-z 25984239 PMC4432772

[36] Smyth GK. Linear models and empirical bayes methods for assessing differential expression in microarray experiments. Stat Appl Genet Mol Biol. 2004; 3:Article3. 10.2202/1544-6115.1027 16646809

[37] Vergoulis T, Vlachos IS, Alexiou P, Georgakilas G, Maragkakis M, Reczko M, Gerangelos S, Koziris N, Dalamagas T, Hatzigeorgiou AG. TarBase 6.0: capturing the exponential growth of miRNA targets with experimental support. Nucleic Acids Res. 2012; 40:D222–29. 10.1093/nar/gkr1161 22135297 PMC3245116

[38] Hsu SD, Tseng YT, Shrestha S, Lin YL, Khaleel A, Chou CH, Chu CF, Huang HY, Lin CM, Ho SY, Jian TY, Lin FM, Chang TH, et al. miRTarBase update 2014: an information resource for experimentally validated miRNA-target interactions. Nucleic Acids Res. 2014; 42:D78–85. 10.1093/nar/gkt1266 24304892 PMC3965058

[39] Xiao F, Zuo Z, Cai G, Kang S, Gao X, Li T. miRecords: an integrated resource for microRNA-target interactions. Nucleic Acids Res. 2009; 37:D105–10. 10.1093/nar/gkn851 18996891 PMC2686554

[40] Li JH, Liu S, Zhou H, Qu LH, Yang JH. starBase v2.0: decoding miRNA-ceRNA, miRNA-ncRNA and protein-RNA interaction networks from large-scale CLIP-Seq data. Nucleic Acids Res. 2014; 42:D92–97. 10.1093/nar/gkt1248 24297251 PMC3964941

[41] Paraskevopoulou MD, Georgakilas G, Kostoulas N, Reczko M, Maragkakis M, Dalamagas TM, Hatzigeorgiou AG. DIANA-LncBase: experimentally verified and computationally predicted microRNA targets on long non-coding RNAs. Nucleic Acids Res. 2013; 41:D239–45. 10.1093/nar/gks1246 23193281 PMC3531175

[42] Paci P, Colombo T, Farina L. Computational analysis identifies a sponge interaction network between long non-coding RNAs and messenger RNAs in human breast cancer. BMC Syst Biol. 2014; 8:83. 10.1186/1752-0509-8-83 25033876 PMC4113672

[43] Zhou M, Wang X, Shi H, Cheng L, Wang Z, Zhao H, Yang L, Sun J. Characterization of long non-coding RNA-associated ceRNA network to reveal potential prognostic lncRNA biomarkers in human ovarian cancer. Oncotarget. 2016; 7:12598–611. 10.18632/oncotarget.7181 26863568 PMC4914307

[44] Xiao Y, Hsiao TH, Suresh U, Chen HI, Wu X, Wolf SE, Chen Y. A novel significance score for gene selection and ranking. Bioinformatics. 2014; 30:801–07. 10.1093/bioinformatics/btr671 22321699 PMC3957066

[45] Jiang W, Mitra R, Lin CC, Wang Q, Cheng F, Zhao Z. Systematic dissection of dysregulated transcription factor-miRNA feed-forward loops across tumor types. Brief Bioinform. 2016; 17:996–1008. 10.1093/bib/bbv107 26655252 PMC5142013

[46] Wang Q, Yu H, Zhao Z, Jia P. EW_dmGWAS: edge-weighted dense module search for genome-wide association studies and gene expression profiles. Bioinformatics. 2015; 31:2591–94. 10.1093/bioinformatics/btv150 25805723 PMC4514922

[47] McKenzie AT, Katsyv I, Song WM, Wang M, Zhang B. DGCA: A comprehensive R package for Differential Gene Correlation Analysis. BMC Syst Biol. 2016; 10:106. 10.1186/s12918-016-0349-1 27846853 PMC5111277

[48] Hou L, Chen M, Zhang CK, Cho J, Zhao H. Guilt by rewiring: gene prioritization through network rewiring in genome wide association studies. Hum Mol Genet. 2014; 23:2780–90. 10.1093/hmg/ddt668 24381306 PMC3990172

[49] Bauer-Mehren A, Rautschka M, Sanz F, Furlong LI. DisGeNET: a Cytoscape plugin to visualize, integrate, search and analyze gene-disease networks. Bioinformatics. 2010; 26:2924–26. 10.1093/bioinformatics/btq538 20861032

[50] Hamosh A, Scott AF, Amberger JS, Bocchini CA, McKusick VA. Online Mendelian Inheritance in Man (OMIM), a knowledgebase of human genes and genetic disorders. Nucleic Acids Res. 2005; 33:D514–17. 10.1093/nar/gki033 15608251 PMC539987

[51] Becker KG, Barnes KC, Bright TJ, Wang SA. The genetic association database. Nat Genet. 2004; 36:431–32. 10.1038/ng0504-431 15118671

[52] Davis AP, Murphy CG, Johnson R, Lay JM, Lennon-Hopkins K, Saraceni-Richards C, Sciaky D, King BL, Rosenstein MC, Wiegers TC, Mattingly CJ. The Comparative Toxicogenomics Database: update 2013. Nucleic Acids Res. 2013; 41:D1104–14. 10.1093/nar/gks994 23093600 PMC3531134

[53] Eppig JT, Blake JA, Bult CJ, Kadin JA, Richardson JE, and Mouse Genome Database Group. The Mouse Genome Database (MGD): comprehensive resource for genetics and genomics of the laboratory mouse. Nucleic Acids Res. 2012; 40:D881–86. 10.1093/nar/gkr974 22075990 PMC3245042

[54] UniProt C. Activities at the Universal Protein Resource (UniProt). Activities at the Universal Protein Resource (UniProt). Nucleic Acids Res. 2014; 42:D191–98. 10.1093/nar/gkt1140 24253303 PMC3965022

[55] Li Y, Qiu C, Tu J, Geng B, Yang J, Jiang T, Cui Q. HMDD v2.0: a database for experimentally supported human microRNA and disease associations. Nucleic Acids Res. 2014; 42:D1070–74. 10.1093/nar/gkt1023 24194601 PMC3964961

[56] Griffiths-Jones S, Grocock RJ, van Dongen S, Bateman A, Enright AJ. miRBase: microRNA sequences, targets and gene nomenclature. Nucleic Acids Res. 2006; 34:D140–44. 10.1093/nar/gkj112 16381832 PMC1347474

[57] Li J, Chen Z, Tian L, Zhou C, He MY, Gao Y, Wang S, Zhou F, Shi S, Feng X, Sun N, Liu Z, Skogerboe G, et al. LncRNA profile study reveals a three-lncRNA signature associated with the survival of patients with oesophageal squamous cell carcinoma. Gut. 2014; 63:1700–10. 10.1136/gutjnl-2013-305806 24522499 PMC4215280

