# Supplementary material for: Systematic analysis of lncRNA and microRNA dynamic features reveals diagnostic and prognostic biomarkers of myocardial infarction
Source: Aging (Albany NY). 2020 Jan 12;12(1):945–64. doi: 10.18632/aging.102667 (PMC6977700; doi:10.18632/aging.102667)
Supplement: Supplementary Figures [file aging-12-102667-s005..pdf]

SUPPLEMENTARY FIGURES

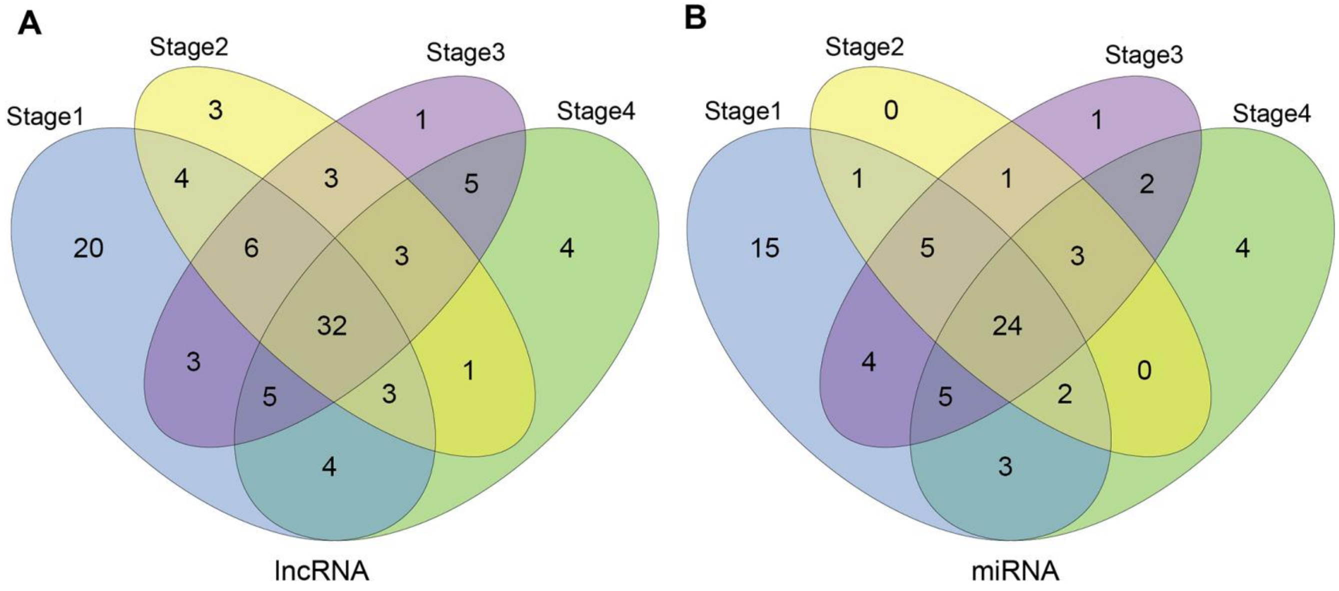

Supplementary Figure 1. Venn diagram of lncRNAs (**A**) and miRNAs (**B**) in dysregulated LmiRM-CTs at four stages during MI progression.

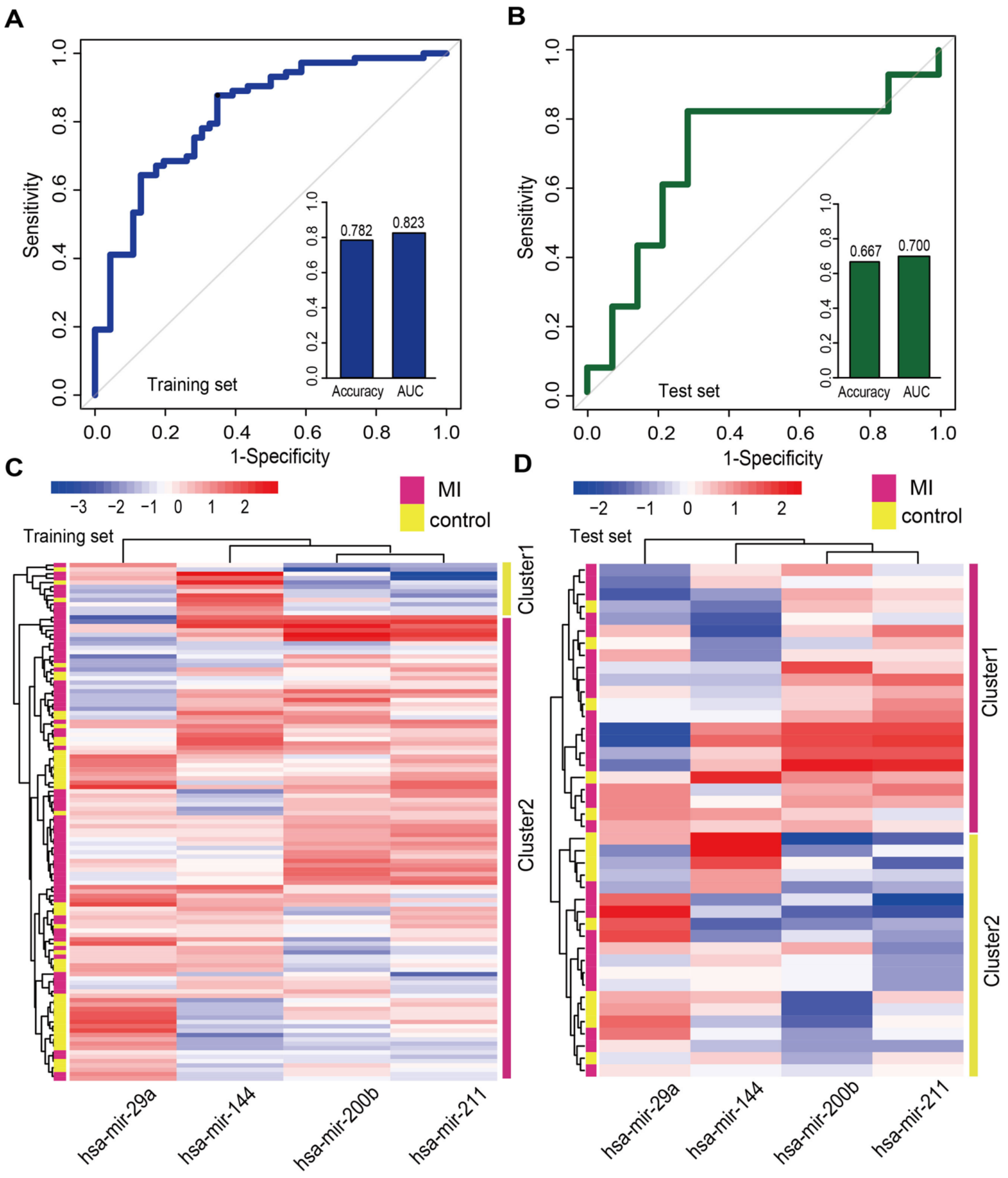

Supplementary Figure 2. Classification performance of miRNA biomarkers for MI diagnosis based on 5-fold cross-validation analysis. Performance evaluation of the 4 diagnostic miRNAs biomarkers in the training (**A**) and test (**B**) sets. Hierarchical clustering heat map of the expression profiles of the 4 miRNAs in (**C**) the training set (119 samples), and (**D**) the test set (42 samples).

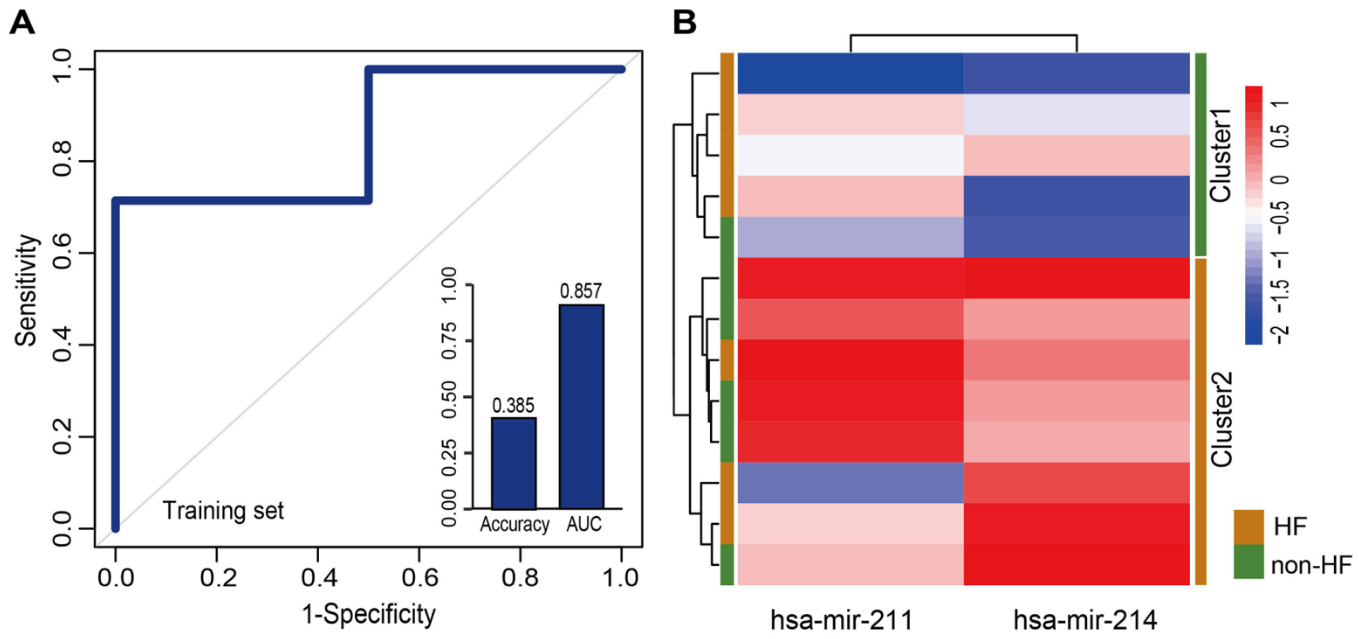

Supplementary Figure 3. Classification performance of miRNA biomarkers for MI prognosis based on 5-fold cross-validation analysis. (**A**) Performance evaluation of the 2 prognostic miRNA biomarkers in the training set. (**B**) Hierarchical clustering heat map of 13 samples based on expression profiles of the 2 miRNAs in the training set.

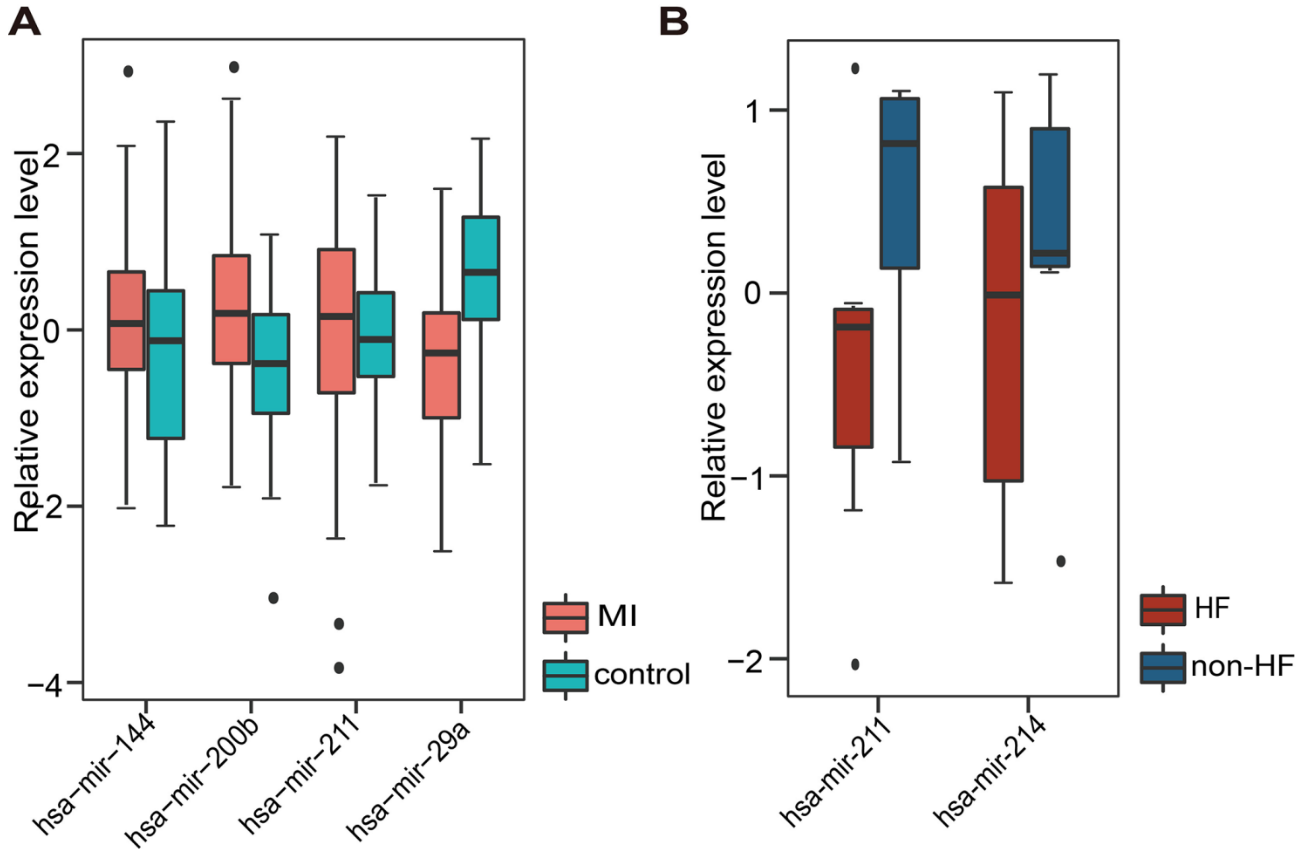

Supplementary Figure 4. Expression levels of diagnostic and prognostic miRNA biomarkers in MI. (**A**) Relative expression of 4 SDE miRNAs in MI and control samples. (**B**) Relative expression of 2 SDE miRNAs in HF and non-HF samples.
