# Supplementary material for: Systematic analysis of lncRNA and microRNA dynamic features reveals diagnostic and prognostic biomarkers of myocardial infarction
Source: Aging (Albany NY). 2020 Jan 12;12(1):945–64. doi: 10.18632/aging.102667 (PMC6977700; doi:10.18632/aging.102667)
Supplement: Supplementary Table 3 [file aging-12-102667-s003..pdf]

Supplementary Table 3. Ranked percentage of SDE transcripts (mRNAs, miRNAs, and lncRNAs) and known MI-related transcripts contained in dysregulated LmiRM-CTs obtained using our method.

	**Top 5%**	**Top10%**	**Top1 5%**	**Top 20%**	**Top 30%**	**Top 40%**	**Top 50%**	**100%**
SDE_transcripts	96.77%	97.14%	96.82%	96.34%	94.86%	93.55%	93.50%	92.35%
MI_ transcripts	8.06%	7.62%	9.55%	8.38%	8.69%	8.06%	7.32%	6.82%
